# *Acremonium terricola* culture plays anti-inflammatory and antioxidant roles by modulating MAPK signaling pathways in rats with lipopolysaccharide-induced mastitis

**DOI:** 10.29219/fnr.v64.3649

**Published:** 2020-11-13

**Authors:** Yang Li, Xin Jiang, Hongjian Xu, Jingyi Lv, Guangning Zhang, Xiujing Dou, Yonggen Zhang, Xiaoxiang Li

**Affiliations:** 1 College of Animal Science and Technology, Northeast Agricultural University, Harbin, China; 2 Microbial Biological Engineering Company Limited, Hefei, China

**Keywords:** Acrermonium terricola culture, mastitis, anti-inflammation, antioxidant

## Abstract

**Background:**

As a major disease affecting dairy cow production worldwide, bovine mastitis is caused by a variety of pathogenic microorganisms that eventually cause mammary gland inflammation. Acremonium terricola culture (ATC) is a new type of affordable feed additive produced by the solid fermentation of A. terricola isolated from *Cordyceps gunnii* and exerted its anti-inflammatory effect.

**Objectives:**

To evaluate the protective effects of ATC on mastitis and investigate its active mechanism, a lipopolysaccharide (LPS)-induced rat mastitis model was used in two experiments.

**Design:**

In Experiment 1, a total of 40 female Sprague–Dawley rats were used to determine the optimal supplementary dose of ATC via gavage trial. In Experiment 2, we examined the effects of an optimal dose of ATC on LPS-induced mastitis in rats.

**Results:**

The results of Experiment 1 showed that administration of ATC improved growth performance and antioxidant functions in the serum and the liver, as well as immunoglobulin A, G, and M levels in rat serum, and it decreased the content of alanine aminotransferase, aspartate aminotransferase, triglyceride, cholesterol, low-density lipoprotein, and serum urea nitrogen in rat serum; a dosage of 250–1,250 mg/kg/day was shown to be high enough to be effective. The results of Experiment 2 indicated that ATC can relieve the inflammatory reaction of mammary glands in rats, and the LPS-induced expression of tumor necrosis factor-α, interleukin-1β, interleukin-6, and inducible nitric oxide synthase significantly decreased after ATC treatment. Moreover, our results demonstrated that ATC markedly enhanced the activity of antioxidase in this rat mastitis model. The results of Western blot analysis revealed that ATC could suppress the expression of toll-like receptor 4, phosphorylation of extracellular signal-regulated kinase, and activity of c-Jun N-terminal kinase in the LPS-stimulated mastitis model.

**Conclusion:**

Taken together, ATC was shown to exert its anti-inflammatory effect by blocking mitogen-activated protein kinase signaling pathways. These results demonstrate that ATC exerts anti-inflammatory and antioxidant effects in mastitis prevention.

## Popular scientific summary

*Acremonium terricola* culture (ATC) is a new type of affordable feed additive. ATC plays anti-inflammatory and antioxidant roles in rats with lipopolysaccharide-induced mastitis. ATC pretreatment plays a role in anti-inflammatory action by interfering with TLR4 expression, which subsequently inhibits the downstream MAPK signaling pathways and the release of the pro-inflammatory cytokines.

As a major disease affecting dairy cow production worldwide, bovine mastitis is caused by a variety of pathogenic microorganisms that eventually cause mammary gland inflammation (1), consequently causing reduced milk yield and milk quality and increased treatment costs, thereby resulting in economic losses for the dairy industry worldwide (2). In addition to maintaining environmental hygiene, the application of exogenous substances is an important measure for preventing mastitis. Chinese medicines, such as *Geniposide* and *Baicalin*, have been extensively used as antibiotic substitutes in the prevention of mastitis (3, 4). *Cordyceps gunnii* has long been used as a health food because of its ‘Yin-nourishing’ and ‘Yang-invigorating’ actions as a famous traditional Chinese herbal medicine. In addition, it has attracted much attention due to the presence of anti-inflammatory, antitumor, and immunomodulatory effects (5). However, due to the scarcity and high price of *C. gunnii*, its development and application in the livestock industry has been limited. Interestingly, *Acremonium terricola* culture (ATC) is a new and affordable type of feed additive produced by the solid fermentation of *A. terricola* isolated from *C. gunnii*, and its active ingredients and functions are similar to those of natural *Cordyceps*. Our previous research found that ATC has a wide range of biological functions, such as antioxidant action, immune regulation, and anti-inflammatory activity (6, 7). It was reported that ATC significantly decreased somatic cell count (SCC) in milk in our previous study (6). It has been verified that cordycepin included in ATC can attenuate inflammatory cytokine expression *in vitro* (8). Therefore, it is theoretically feasible for ATC to prevent and treat bovine mastitis.

To make further our understanding of the protective effects of ATC against mastitis, the active mechanism of action of ATC on mammary glands should be studied in more detail using a mastitis model. It is well-known that *Escherichia coli* is a major pathogen for mastitis. The injection of lipopolysaccharide (LPS), as the critical pathogenic factor of *E. coli*, into a lactating mouse mammary gland via the nipple has been demonstrated to lead to the same syndrome as naturally occurring mastitis caused by *E. coli* (9). Therefore, in this experiment, a rat mastitis model was established by *E. coli* LPS breast injection to perform an ATC anti-inflammatory test. Ideally, such experimental infection studies are conducted using dairy cows. Unfortunately, these bovine studies are expensive and labor intensive. For those reasons, a mouse mastitis model was developed and characterized (10) and has since been successfully used for research and to prevent inflammation (11).

It is well-known that Toll-like receptor 4 (TLR4) is indispensable for LPS signaling (12). The TLR4–LPS interaction leads to the rapid and coordinated activation of the mitogen-activated protein kinase (MAPK) signaling pathway, which includes extracellular signal-regulated kinases (ERK), c-Jun N-terminal kinases (JNK), and p38 kinases, and activates inducible nitric oxide synthase (iNOS) and other pro-inflammatory genes. However, to date, there remain gaps in the understanding of the metabolic pathway of ATC in the treatment of mastitis. Therefore, it was hypothesized that ATC could exert its anti-inflammatory effect by regulating MAPK signaling pathways. To validate the hypothesis of this experiment, two gavage trials were conducted to evaluate the protective effect of ATC against LPS-induced mastitis and to clarify its anti-inflammatory mechanism.

## Materials and methods

### Preparation of ATC

*A. terricola* is a fungus isolated from the sclerotium of *C. gunnii* (13). The fungus is deposited under the China Microbial Culture Collection at CGMCC No. 0346. ATC is obtained by artificial solid-state fermentation of the strain by Hefei Micro Biological Engineering Co. Ltd. (Hefei, China). The fermentation method was carried out as follows: *A. terricola* was inoculated to a solid medium and cultured at a relative humidity of 80–90% at 25°C for 76 h. Subsequently, it was dried at 80°C for 1 h. The dried sample was passed through a 0.15 mm sieve. The solid fermentation medium consisted of 69.9% corn, 20% soybean meal, 10% wheat bran, 0.08% KH_2_PO_4_, and 0.02% MgSO_4_.

ATC contained the following functional ingredients: cordycepin (3′-deoxyadenosine; 0.0432% of dry matter [DM]), cordycepic acid (d-mannitol; 8.45% of DM), *Cordyceps* polysaccharide (galactomannan; 4.46% of DM), ergosterol (0.0597% of DM), and total amino acid content (21.81% of DM). It contained 63.37% nitrogen-free extract, 24.53% crude protein (CP), 5.6% moisture, 5.0% crude fiber (CF), 4.04% crude ash, and 3.06% ether extract (EE).

### Animals and housing conditions

The two experiments were conducted at the Experimental Practice and Demonstration Center of Northeast Agricultural University (Harbin, China). The Sprague–Dawley (SD) rats (40 females used in first experiment; 40 females and 40 males used in second experiment) and the base feed were purchased from Beijing Vital River Laboratory Animal Technology Co., Ltd. (Beijing, China), and all experimental procedures were approved by the Northeast Agricultural University Animal Science and Technology College Animal Care and Use Committee (protocol number: NEAU-[2011]-9). The rats were housed in room with free access to rodent chow and water and were kept in an environment with a controlled temperature (23 ± 1°C) and humidity (60 ± 10%) under a 12 h light/dark cycle. All animal experiments were started after 1 week of acclimation.

### Experimental design

#### Experiment 1

A total of 40 female SD rats (7 weeks old, 165.93 ± 5.64 g) were randomly divided into five groups consisting of eight rats each (treatment group: gastric perfusion of ATC (10, 50, 250, and 1,250 mg/kg body weight [BW]); control group: gastric perfusion of saline) for 21 days gavage trial.

#### Experiment 2

The 40 female rats (7 weeks old, 165.2 ± 4.82 g) were randomly divided into a control group (control), LPS group (LPS), LPS + ATC group (LPS + ATC), and ATC group (ATC). This experiment lasted until 1 week after the birth of their offspring. A female rat and a male rat were kept in one cage for mating for 4 weeks after the experiment began. Throughout the trial period, the rats in LPS + ATC and ATC groups were administered physiological saline (5 mL) with ATC (250 mg/kg BW) by gavage, seven times per week. Meanwhile, rats in control and LPS groups were administered equal volumes of physiological saline that lacked ATC. The rat mastitis model was established by the injection of LPS (0.2 mg/mL LPS 50 µL) into the mammary ducts of rats from LPS and LPS + ATC on day 7 after the birth of their offspring. Meanwhile, the rats from control and ATC received 50 µL of sterile PBS via mammary duct injection.

### Sample collection

#### Experiment 1

The feed intake was monitored every day for individual rats by weighing the amounts of feed offered and refused daily, BW was measured every 5 days, and then the rats were sacrificed at the end of the study via eyeball extraction (14). Blood was taken from the retro-orbital plexus on day 21 and then centrifuged at 1,200 × *g* for 10 min; then, the serum was prepared and stored at −20°C for further analysis. After sacrifice, liver sections were immediately excised and rinsed in normal saline. Thereafter, liver tissue homogenates (10%) were prepared in chilled normal saline and were then centrifuged at 2,000 × *g* at 4°C for 10 min; thereafter, the supernatants were frozen and preserved at −20°C until evaluation for antioxidant function.

#### Experiment 2

The pups were removed from the lactating rats 1 h before the injection of LPS. The rats were sacrificed at 24 h after LPS was injected and mammary glands were observed and harvested. One tube of the mammary gland tissue fixed in 10% formalin was kept at 4°C for further histological analysis, and another tube of mammary gland tissue was stored at −80°C to detect cytokine production, antioxidant indices, iNOS and TLR4 expressions, and the MAPK signaling pathway.

### Chemical analyses

#### Nutrient composition of ATC

The contents of ergosterol, cordycepin, and cordycepic acid, and *Cordyceps* polysaccharide in ATC were determined by high-performance liquid chromatography (15). The amino acid content was determined using a HITACHI L-8800 automatic amino acid analyzer (HITACHI Co., Tokyo, Japan). The DM (method 930.15), CF (method 993.21), crude ash (method 942.05), EE (method 920.39), and CP (method 976.05) were analyzed according to the methods of the Association of Official Analytical Chemists (16).

#### Experiment 1

The serum total protein, alkaline phosphatase, urea nitrogen, alanine transaminase (ALT), aspartate transaminase (AST), cholesterol, triglyceride, high-density lipoprotein, and low-density lipoprotein levels were analyzed with a fully automatic biochemical analyzer using standard commercial kits supplied from Biosino Biotec (Beijing, China). The concentrations of immunoglobulin A, M, and G in the serum were analyzed using enzyme-linked immunosorbent assay (ELISA) kits (R&D Systems, Minneapolis, MN, USA). The catalase, glutathione peroxidase, total superoxide dismutase, total antioxidant capacity, and malonaldehyde levels in the serum and liver tissue were determined by colorimetry using standard commercial kits (Nanjing Jian Cheng Bioengineering Institute, Nanjing, China). The enzyme activity and total antioxidant capacity levels were expressed as units/mg of protein.

Experiment 2The samples of mammary gland in each group were fixed in formaldehyde for 24 h, and then the fixed tissues were embedded in paraffin, sliced and stained with hematoxylin and eosin (H&E). The histopathological changes of mammary gland tissues were observed by microscope (Olympus, Japan).

The mRNA levels of interleukin-1β (IL-1β), tumor necrosis factor-α (TNF-α), and interleukin-6 (IL-6) in mammary glands were measured using quantitative real-time PCR (qRT-PCR). Total RNA was extracted using Trizol according to the manufacturer’s instructions. Then, cDNA was synthesized according to the manufacturer’s instructions (RR037, Takara, Japan) (16). qRT-PCR was performed to measure the levels of mRNA using the SYBR Green Plus reagent kit (RR420, Takara, Japan) and a 7500 Fast Real-time PCR System (Applied Biosystems, USA) (10). The PCR primer sets used are shown in Table 1. Relative gene expression of mRNA was calculated using the 2^−∆∆Ct^ method, with β-actin as the reference gene (17).

**Table 1:** Nucleotide sequences of primers for RT-PCR

Gene	Forward primer (from 5’ to 3’)	Fragment length (bp)	Genbank no.
	Reverse primer (from 5’ to 3’)		
IL-1β	AAAAATGCCTCGTGCTGTCT	118	NM_031512.2
	TCGTTGCTTGTCTCTCCTTG		
IL-6	AGTTGCCTTCTTGGGACTGA	102	NM_012589.2
	ACTGGTCTGTTGTGGGTGGT		
TNF-α	GTCGTAGCAAACCACCAAGC	147	NM_012675.3
	GAAGAGAACCTGGGAGTAGATAAGG		
β-Actin	GCTCTCTTCCAGCCTTCCTT	101	NM_031144.3
	AGGTCTTTACGGATGTCAACG		

RT-PCR, real-time PCR.

To determine the production of cytokines more accurately, the content of pro-inflammatory cytokines was determined by ELISA. Total protein of mammary gland tissues was extracted using the M-PER Mammalian Protein Extraction Reagent (Thermo Scientific, Waltham, MA, USA). The concentration of protein was measured using the Bicinchoninic Acid (BCA) method. The levels of TNF-α, IL-6, and IL-1β in mammary glands were measured using ELISA (BOSTER, Wuhan, China) according to the manufacturer’s recommendations. The mammary gland tissues were homogenized in PBS (1:9, w/v). Afterward, the homogenate was centrifuged twice at 2,000 × *g* to remove the lipids. In addition, total antioxidant capacity, glutathione peroxidase, total superoxide dismutase, malonaldehyde, and catalase levels in mammary glands were measured by colorimetry using standard commercial kits (Nanjing, China).

For the Western blot analysis, 50 μg samples of denatured protein lysates were loaded on polyacrylamide gels (10%) and transferred onto Polyvinylidene Fluoride (PVDF). membranes. Then, the membranes were blocked with 5% skimmed milk in Tris Buffered Saline with Tween (TBST) for 2 h at 37°C before incubation with primary antibodies in TBST overnight at 4°C in 1% BSA. Reprobing with a secondary Horseradish Peroxidase (HRP)-conjugated antibody was performed for 1 h at room temperature. The antibodies used were anti-p38 MAPK, anti-p-p38 MAPK, anti-ERK, anti-p-ERK, anti-JNK, anti-p-JNK, anti-TLR4, and anti-iNOS, and β-actin was used as a control. Finally, blots were visualized using the ECL plus Western blot detection system. The expression of each protein was normalized to β-actin. The images were captured using an ImageLab developing system (DNR Bio-Imaging Systems, Jerusalem, Israel).

### Statistical analysis

Data were analyzed using ANOVA with SAS software, and Duncan’s multiple range test was used for both experiments. The data are expressed as means ± standard error of the mean, and statistical significance was defined at *P* < 0.05.

## Results

### Effects of ATC on growth performance in rats

**Fig. 1:**
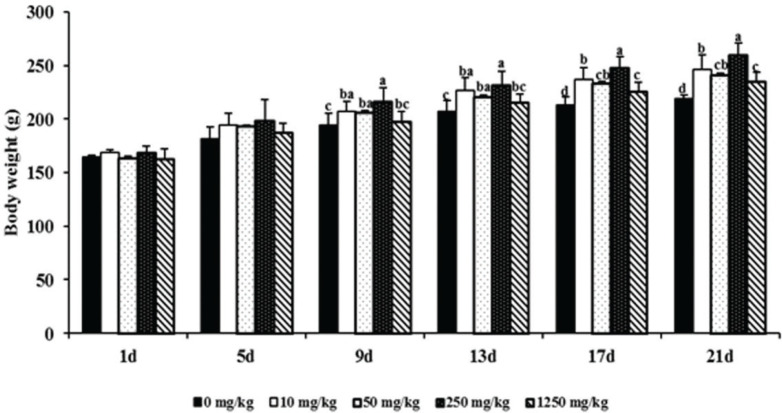
Effects of *Acremonium terricola* culture (ATC) on body weight (BW) in SD rats. 0 mg/kg = gastric perfusion of saline; 10 mg/kg = gastric perfusion of 10 mg/kg BW ATC; 50 mg/kg = gastric perfusion of 50 mg/kg BW ATC; 250 mg/kg = gastric perfusion of 250 mg/kg BW ATC; 1,250 mg/kg = gastric perfusion of 1,250 mg/kg BW ATC. Means with different letters within the same row differ significantly (*P* < 0.05). The error bars represent the SD (*n* = 8 rats/group).

**Fig. 2:**
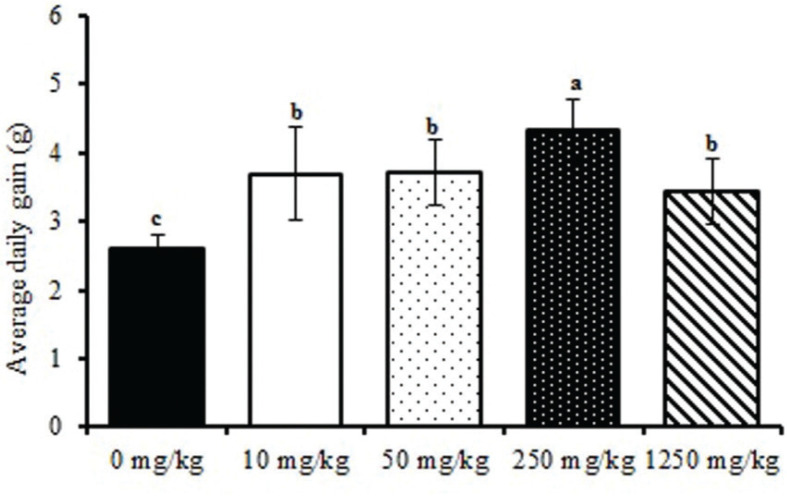
Effects of ATC on average daily gain in SD rats. 0 mg/kg = gastric perfusion of saline; 10 mg/kg = gastric perfusion of 10 mg/kg BW ATC; 50 mg/kg = gastric perfusion of 50 mg/kg BW ATC; 250 mg/kg = gastric perfusion of 250 mg/kg BW ATC; 1,250 mg/kg = gastric perfusion of 1,250 mg/kg BW ATC. Means with different letters within the same row differ significantly (*P* < 0.05). The error bars represent the SD (*n* = 8 rats/group).

There is no difference among the treatments for dry matter intake (data not shown). Additionally, as shown in Fig. 1, no significant differences were found in BW (*P* = 0.14) at the start of the experiment. The BWs of the treatment group from day 9 were higher than those of the control group, and the highest BW was seen (*P* < 0.01) in the 250 mg/kg treatment group. Compared with the control group, ATC supplementation markedly increased average daily gain (*P* < 0.0001), the highest average daily gain was seen in the 250 mg/kg treatment, and average daily gain was not significantly different among the other test groups (Fig. 2).

### Effects of ATC on serum biochemical and immune indicators in rats

Serum parameters are shown in Table 2. Compared with the control group, the activity of AST (*P* = 0.0004) and ALT (*P* < 0.0001), and contents of cholesterol (*P* = 0.0005), SUN (*P* = 0.0002), low-density lipoprotein (*P* < 0.0001), and triglyceride (*P* = 0.006) were significantly reduced in rats that received ATC treatment. In contrast, the higher dose of ATC appeared to significantly induce higher levels of immunoglobulins A, G, and M (*P* < 0.0001).

**Table 2:** Effects of *Acremonium terricola* culture on serum biochemical parameters in rats

Item	Dose					SEM	*P*
	0 mg/kg BW	10 mg/kg BW	50 mg/kg BW	250 mg/kg BW	1,250 mg/kg BW		
TP (g/L)	74.75	72.50	76.75	75.25	74.00	1.18	0.16
ALT (IU/L)	50.63^a^	39.25^cb^	36.25^cd^	32.50^d^	42.75^b^	1.80	<0.0001
AST (IU/L)	192.13^a^	153.00^b^	168.25^b^	160.00^b^	165.25^b^	5.71	0.0004
TG (mmol/L)	0.95^a^	0.95^a^	0.82^b^	0.77^b^	0.87^ba^	0.04	0.006
CHOL (mmol/L)	2.37^a^	1.75^b^	1.87^b^	1.78^b^	1.87^b^	0.10	0.0005
HDL (mmol/L)	0.71	0.70	0.74	0.67	0.71	0.02	0.43
LDL (mmol/L)	0.30^a^	0.20^b^	0.23^b^	0.19^b^	0.22^b^	0.01	<0.0001
SUN (mmol/L)	7.00^a^	6.28^ba^	5.73^bc^	5.30^c^	5.48^c^	0.25	0.0002
AKP (IU/L)	158.88	154.00	160.25	167.25	158.50	3.29	0.0992
IgA (µg/mL)	28.38^c^	28.84^c^	30.91^b^	34.77^b^	35.88^a^	0.70	<0.0001
IgG (µg/mL)	275.47^d^	285.45^d^	315.17^c^	377.79^a^	363.57^b^	4.87	<0.0001
IgM (µg/mL)	7.51^d^	7.75^cd^	8.62^cb^	10.15^a^	9.09^b^	0.30	<0.0001

a–d Within row means with different superscripts are significantly different at *P* < 0.05.

TP: total protein; AKP: alkaline phosphatase; SUN: serum urea nitrogen; ALT: alanine transaminase; AST: aspartate transaminase; CHOL: cholesterol; TG: triglyceride; HDL: high-density lipoprotein; LDL: low-density lipoprotein; IgA: immunoglobulin A; IgM: immunoglobulin M; IgG: immunoglobulin G.

### Effects of ATC on antioxidant capacity in the serum and liver

**Fig. 3:**
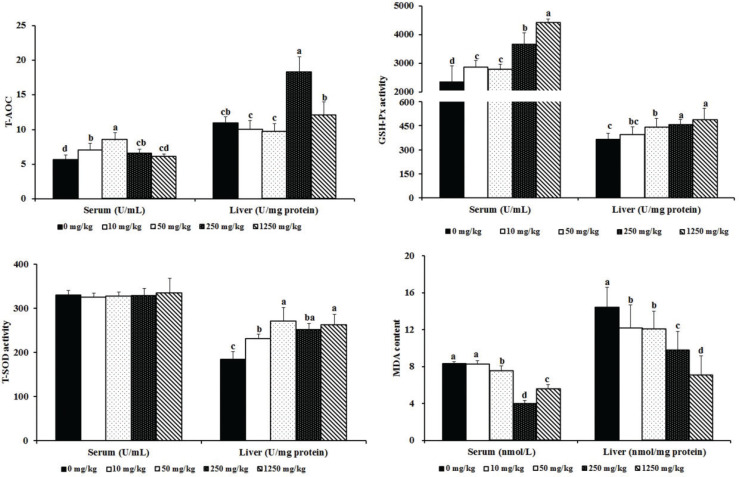
Effects of ATC on antioxidant functions in SD rats. 0 mg/kg = gastric perfusion of saline; 10 mg/kg = gastric perfusion of 10 mg/kg BW ATC; 50 mg/kg = gastric perfusion of 50 mg/kg BW ATC; 250 mg/kg = gastric perfusion of 250 mg/kg BW ATC; 1,250 mg/kg = gastric perfusion of 1,250 mg/kg BW ATC. Means with different letters within the same row differ significantly (*P* < 0.05). The error bars represent the SD (*n* = 8 rats/group).

The ATC significantly increased antioxidase activity and decreased malonaldehyde concentration in the serum and liver (Fig. 3). Compared with the control group, ATC had no effects on total superoxide dismutase activity in the serum or catalase activity (*P* > 0.05). However, ATC supplementation significantly increased the activity of total superoxide dismutase in the liver (*P* < 0.0001) and glutathione peroxidase (*P* < 0.0001), increased the total antioxidant capacity level (*P* < 0.0001), and decreased malonaldehyde content (*P* < 0.0001) in the serum and liver; the most robust effects were seen in the 250–1,250 mg/kg groups.

### Effects of ATC on anatomy map and histopathological changes in rat mastitis model

**Fig. 4:**
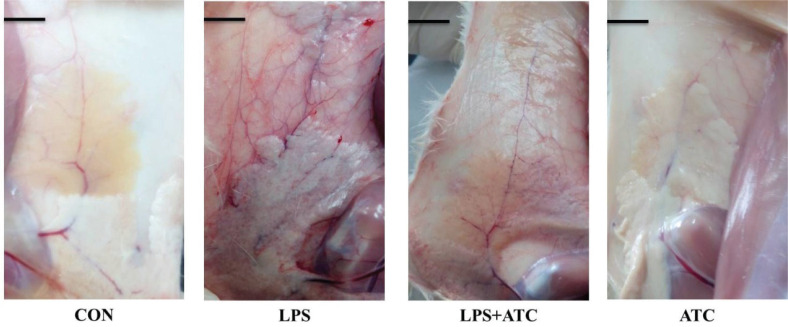
Anatomy maps of mammary glands in rats. Twenty-four hours after the infusion of LPS, anatomy maps of mammary glands in rats were observed. CON = control group; LPS = LPS group; LPS + ATC = LPS + ATC group (250 mg/kg BW by gavage); ATC = ATC group (250 mg/kg BW by gavage). Scale bars, 1 cm.

**Fig. 5:**
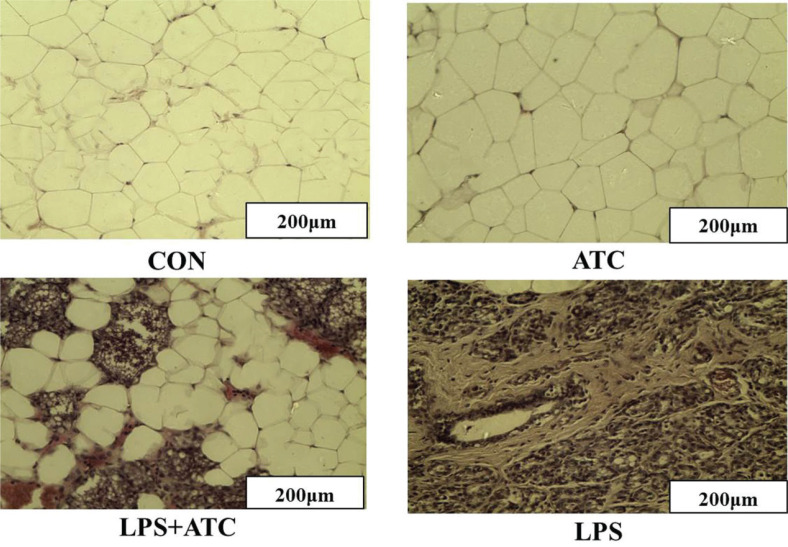
Effects of ATC on histopathological changes in LPS-induced mammary glands. Twenty-four hours after the infusion of LPS, the histopathology of mammary glands was observed by H&E staining. CON = control group; LPS = LPS group; LPS + ATC = LPS+ ATC group (250 mg/kg BW by gavage); ATC = ATC group (250 mg/kg BW by gavage).

As shown in Figs. 4 and 5, normal anatomic formation of mammary tissue in the control and ATC groups was observed. In the control and ATC groups, no pathologic changes were observed in the mammary glands. However, in the LPS group, we found obvious inflammatory response in mammary glands, such as red and swollen tissue, and the mammary glands were infiltrated with multiple inflammatory cells. In the LPS+ATC group, the LPS-induced pathological changes and inflammatory cell infiltration in the mammary glands were significantly ameliorated compared with the LPS group. The structural integrity of the mammary gland cells was obviously restored.

### Effects of ATC on cytokine production

**Fig. 6:**
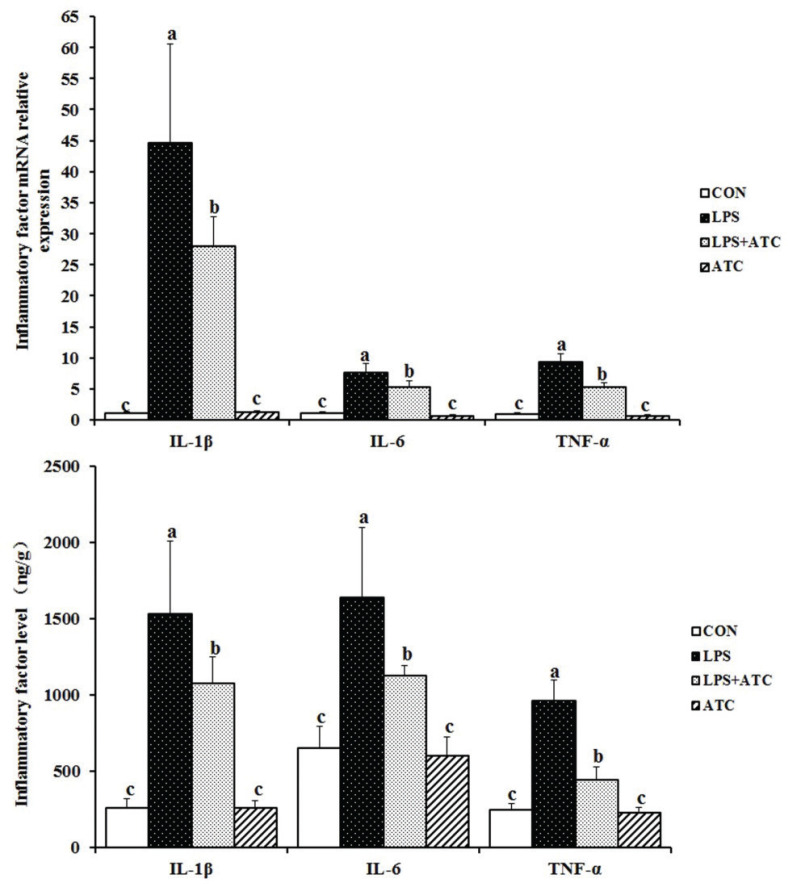
Effects of ATC on cytokine production in LPS-induced mammary glands. RT-PCR and ELISA were used to measure the relative mRNA expression and production of cytokines in the mammary gland homogenates. CON = control group; LPS = LPS group; LPS + ATC = LPS+ ATC group (250 mg/kg BW by gavage); ATC = ATC group (250 mg/kg BW by gavage). Means with different letters within the same row differ significantly (*P* < 0.05). The error bars represent the SD (*n* = 10 rats/group).

As shown in Fig. 6, after LPS stimulation, the contents of TNF-α, IL-1β, and IL-6 explosively increased. Compared with the LPS group, ATC reduced the production of TNF-α, IL-1β, and IL-6 in the LPS+ATC group. No significant changes were seen in the control and ATC groups. The qRT-PCR data were consistent with the results of ELISA experiments.

### Effects of ATC on antioxidant function

**Fig. 7:**
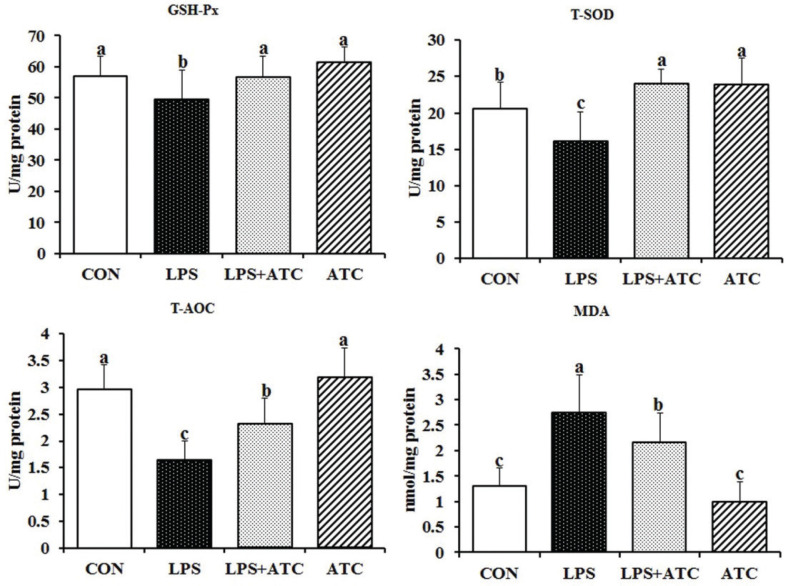
Effects of ATC on antioxidant functions in LPS-induced mammary gland. CON = control group; LPS = LPS group; LPS + ATC = LPS+ ATC group (250 mg/kg BW by gavage); ATC = ATC group (250 mg/kg BW by gavage). Means with different letters within the same row differ significantly (*P* < 0.05). The error bars represent the SD (*n* = 10 rats/group).

We observed that antioxidant functions were significantly lower in the LPS group and increased with higher ATC intake levels, although The total antioxidant capacity (T-AOC). was still lower than in the control group (Fig. 7).

### Effects of ATC on the expression of LPS-induced iNOS

**Fig. 8:**
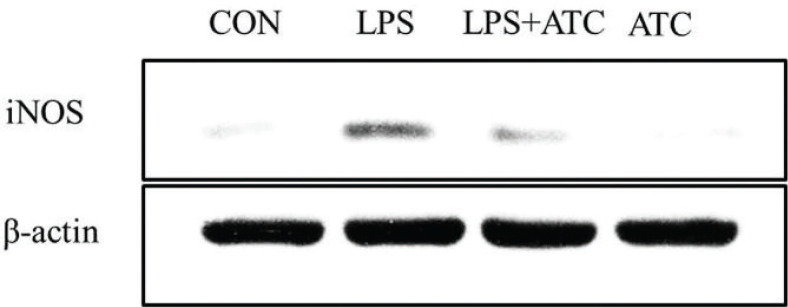
Effects of ATC on iNOS expression in LPS-induced mammary gland. Mammary tissue was then prepared, and Western blots were performed using an antibody specific for murine iNOS. CON = control group; LPS = LPS group; LPS + ATC = LPS+ ATC group (250 mg/kg BW by gavage); ATC = ATC group (250 mg/kg BW by gavage); (*n* = 10 rats/group).

The effect of ATC on the expression of LPS-induced iNOS was studied. Fig. 8 shows the results of iNOS expression in mammary glands. The ATC supplement decreased LPS-induced iNOS expression in the LPS+ATC group compared with the LPS group.

### Effects of ATC on the expression of TLR4

**Fig. 9:**
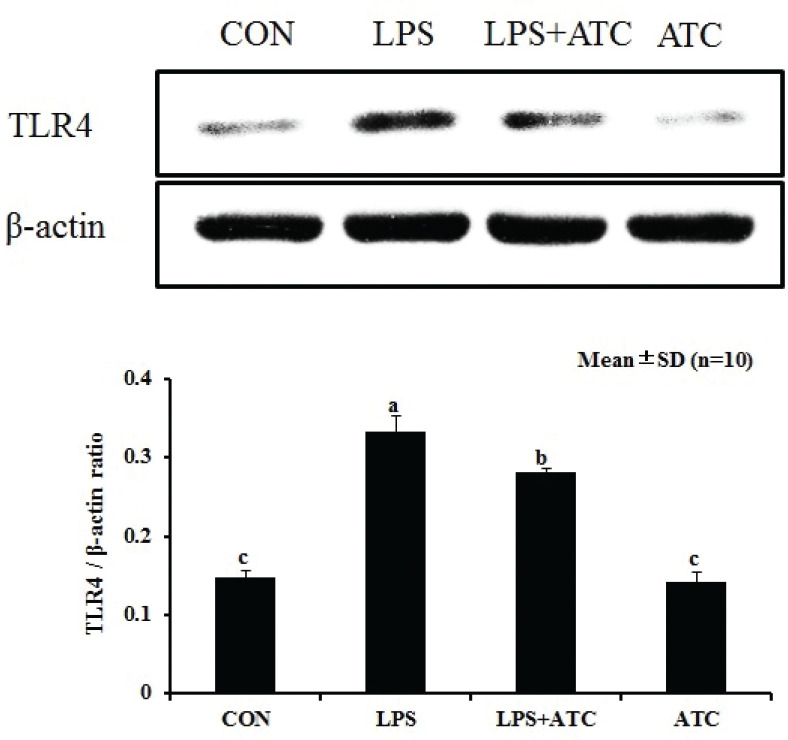
Effects of ATC on the expression of TLR4 in LPS-induced mammary glands. MAPK protein samples were analyzed by Western blot with specific antibodies. β-Actin was used as a control. Means with different letters within the same row differ significantly (*P* < 0.05). The error bars represent the SD. CON = control group; LPS = LPS group; LPS + ATC = LPS + ATC group (250 mg/kg BW by gavage); ATC = ATC group (250 mg/kg BW by gavage).

The expression of TLR4 was observed in the mammary gland tissue (Fig. 9). After stimulation with LPS, Western blot analysis showed that the expression of TLR4 was markedly higher than in the control group but was downregulated in the LPS+ATC group.

### Effects of ATC on the MAPK signaling pathways

**Fig. 10:**
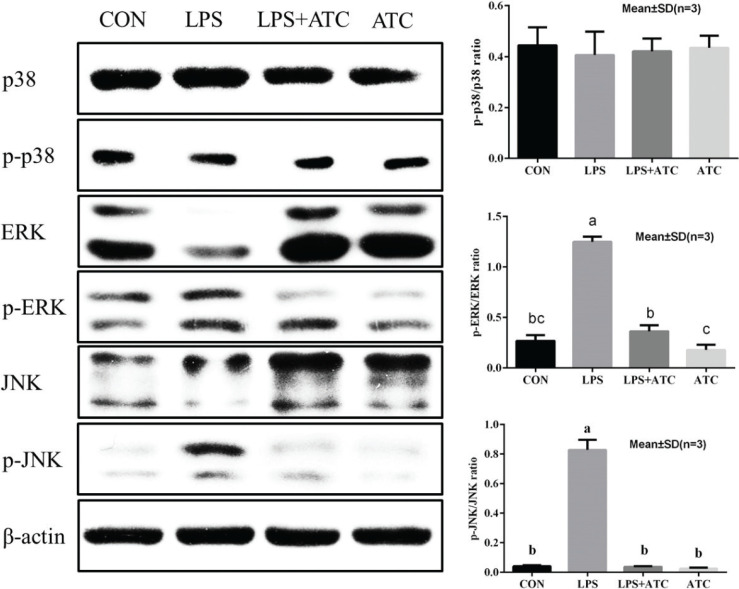
Effects of ATC on the expression of MAPKs in LPS-induced mammary glands. MAPK protein samples were analyzed by Western blot with specific antibodies. β-Actin was used as a control. Means with different letters within the same row differ significantly (*P* < 0.05). The error bars represent the SD. CON = control group; LPS = LPS group; LPS + ATC = LPS + ATC group (250 mg/kg BW by gavage); ATC = ATC group (250 mg/kg BW by gavage).

As shown in Fig. 10, MAPK signal pathways were activated by LPS, and the phosphorylation levels of ERK and JNK were markedly increased. However, compared with the LPS group, ATC pretreatment significantly inhibited the LPS-induced increase in ERK and JNK phosphorylation in the ATC+LPS group.

## Discussion

For the first time, we demonstrated that ATC improves the productivity and health of rats. The reason ATC supplementation can enhance growth performance and health is mainly due to the presence of specific compounds, such as cordycepic acid, *Cordyceps* polysaccharide, and cordycepin. ATC and *Cordyceps* substances containing the same active ingredients are known as *Cordyceps* feed additive due to similar biological function. Furthermore, many studies have reported that *Cordyceps* substances and active ingredients have the ability to improve growth performance and antioxidant function. The improvement of growth performance in broiler chicks (18) and laying hens (19) after feeding with fermentation products and the by-product of *Cordyceps militaris*, and the enhancement of BW and feed conversion rate of white shrimp (20) after supplementing with *Cordyceps* polysaccharides suggest that *Cordyceps* substances and active ingredients are effective in applications for animal husbandry. Additionally, our previous study showed that ATC supplementations accelerated the BW gain of calves, increased the BW and feed efficiency, and improved antioxidant and immune functions (7), which are results similar to those of the current study. The mechanism for the growth performance improvement of rats may be attributed to ATC increasing the feed conversion ratio (20). The BW gain of rats might also be related to the antibacterial action of functional ingredients of ATC. Previous studies have demonstrated that cordycepin and *Cordyceps* polysaccharides have inhibitory effects on bacteria (21, 22). Studies with broiler chicks have demonstrated that extracts of mycelia from *Cordyceps sinensis* inhibited pathogenic bacterial growth and increased the population of *Lactobacillus* spp. in the small intestine, which, in turn, improved BW gain (23). Therefore, the antibacterial action and probiotic function of the active ingredient in ATC are key factors in the improvement of growth performance. The objective of this trial was to determine the optimal dose for subsequent testing, so intestinal flora was not detected. Therefore, this topic still requires further study to illuminate the influence of ATC on intestinal microbial population and digestive metabolism.

Serum biochemical indicators comprehensively reflect body status and are the most sensitive and direct indicators mirroring the health condition of rats. The serum total protein, high-density lipoprotein, and alkaline phosphatase levels were not influenced by ATC addition. ALT and AST levels are important parameters, which indicate the status of hepatic cells. Previous studies have demonstrated that *C. sinensis* mycelial powder (24) and extract of *C. militaris* (25) are able to improve antioxidant function in rats, inhibit the secretion of pro-inflammatory factors in rats, and reduce the activities of AST and ALT. In the current study, decreases in the AST and ALT contents suggest that ATC may have protective effects on hepatic cells, which may be related to the antioxidant and anti-inflammatory effects of the active ingredients in ATC.

High levels of blood triglyceride, cholesterol, and low-density lipoprotein are responsible for cardiovascular diseases (26). In the current study, a very significant decrease was observed in the levels of low-density lipoprotein, triglyceride, and cholesterol, strengthening the lipid-lowering activity and illustrating the cardio-protective effects of ATC. Kiho et al. suggested that the levels of blood triglyceride and cholesterol in normal and diabetic rats decreased in the treatment group at 3 or 6 h after the addition of *Cordyceps* polysaccharide (27). Park et al. also observed that the addition of *C. militaris* to diabetic rats resulted in apparent decrease in the levels of triglyceride, total cholesterol, and low-density lipoprotein-cholesterol (28). Based on our study, increases in functional components (*Cordyceps* polysaccharide, cordycepic acid, and cordycepin) are vital in preventing cardiovascular and cerebrovascular diseases, but the reason for this is still unclear. Therefore, further research is needed to explore the underlying mechanism of disease prevention. SUN is one of the important indicators, which reflects renal function. An increasing level of urea nitrogen in the serum suggests the kidneys may be damaged. Research shows that a Chinese herb can reduce nephrotoxicity and decrease SUN content (29). Li et al. found that addition of *C. sinensis* markedly decreased the total cholesterol content and reduced the levels of ALT and urea nitrogen in the serum of patients, which is consistent with our results (30). Immunoglobulins are proteins that bind to foreign agents, such as bacteria and viruses, and help to eliminate them from the body, thereby leading to complement activation and neutralization of toxins. Our results indicated that administration of ATC greatly increases the contents of immunoglobulin A, G, and M. There is a great deal of research that shows that the active ingredients and extracts of *Cordyceps* improved the concentrations of immunoglobulins and enhanced the humoral immune function in rats (31–33), agreeing with our findings.

Total antioxidant capacity is a single measurement that describes the dynamic equilibrium between prooxidants and antioxidants. Free-radical-induced lipid peroxidation is an important consequence of oxidative stress and has been associated with a number of diseases. Malonaldehyde is often used as an indicator of oxidative stress and is also a product of lipid peroxidation. Therefore, measuring these indices may help us understand the antioxidant function of rats. Active ingredients of cultured *C. sinensis* exert potent antioxidant and anti-lipid peroxidation functions and can be effective in scavenging various types of oxygen-free radicals and their products (20, 34, 35). Research has demonstrated that cordycepin may be a promising candidate for the prevention of alcohol-induced hepatotoxicity (36), and *C. sinensis* shows great potential for preventing hepatinica (24, 37, 38). Using both serum and tissue research in rats, Liu et al. concluded that *C. militaris* polysaccharides can improve the antioxidant capacity of the liver and reduce the malonaldehyde content in the serum, similar to our results (32). Additionally, ATC demonstrated its strong antioxidant function by significantly improving antioxidase activity and decreasing SCC in calves and dairy cows (6, 7). These positive results, already reported in a variety of studies (24, 28, 39), only demonstrated the function of the active ingredient in *Cordyceps*, whereas the research on its antioxidative mechanisms is lacking. This is why further study is required to determine its mode of action. After analyzing our results, we found many favorable outcomes regarding growth performance and serum biochemical indicators in the 250 mg/kg group. Therefore, subsequent research in Experiment 2 was conducted with this dosage.

Mastitis is a common multiple disease in dairy cows. As the main cause of considerable economic losses, prevention and treatment of mastitis is critical for the dairy industry (2). However, because there are many disadvantages of experiments using cows, such as difficulty and expense, over the last few years, the mastitis model has been used to prevent inflammation extensively (3). Previous studies have demonstrated that ATC significantly decreased SCC in milk and increased milk production of dairy cows (6). Therefore, we think that ATC may have very good protective effects against mastitis. In the current study, *E. coli* LPS caused redness and swelling of nipples, and the LPS-induced pathological changes and the infiltration of inflammatory cells in the mammary glands were significantly ameliorated by administration with ATC. This astonishing phenomenon confirmed that ATC has potent anti-inflammatory activity.

To understand further anti-inflammatory mechanisms of ATC, the rat was chosen as a model for studying antioxidant levels, pro-inflammatory factor regulation, and signal transduction pathways. ATC possesses important anti-inflammatory and immune functions due to the presence of specific compounds. *A. terricola* was isolated from *C. gunnii*. The active components (cordycepin, ergosterol, *Cordyceps* polysaccharide, and cordycepic acid) of ATC are similar to those of natural *C. gunnii*. The role of the active ingredients of *Cordyceps* in the regulation of cytokines and in antioxidant function has been demonstrated by many experiments (32, 35, 39). Therefore, based on previous research results, we assumed that *Cordyceps* polysaccharide in ATC is possibly involved in the antioxidant function of ATC in mammary glands. Research has demonstrated that *C. militaris* polysaccharides can enhance antioxidation ability in immunosuppressed rats by increasing antioxidant enzyme activities and decreasing advanced lipoxidation end product contents *in vivo* (35, 40). Some of the many animals in which *Cordyceps* polysaccharide and ATC have been found to strengthen antioxidant functions include calves (7), cows (6), rats (35), and aquatic animals (20). It is well known that inflammatory injury can accompany oxidative stress (40). Therefore, that *Cordyceps* polysaccharides possess a double effect of antioxidation, and anti-inflammation is critical to ATC-enhanced immunity.

Studies have shown that during pathogen infection, pro-inflammatory cytokines play an important role in the inflammatory response in mastitis (41). A reduction in the levels of pro-inflammatory cytokines indicates that ATC exerts a protective effect on organs under excessively inflammatory reactions (42). In the current study, we measured the levels of pro-inflammatory cytokines using RT-PCR and ELISA methods. Both RT-PCR and ELISA showed that the levels of three pro-inflammatory cytokines in the LPS group were significantly higher than those in the control group but significantly lower than in the LPS+ATC group. Therefore, ATC exerts an anti-inflammatory effect by inhibiting the release of TNF-α, IL-1β, and IL-6. Similarly, addition of ATC possibly changes the dynamics of infiltration of other immune cells into the inflamed area, mainly by reducing the secretion of pro-inflammatory cytokines. In addition, we think that the anti-inflammatory effect of ATC is dependent on cordycepin. One study found that cordycepin showed significant anticancer, anti-inflammatory, pharmacological, and immune stimulation effects (8). Moreover, after LPS stimulation, cordycepin inhibited the expression of nitric oxide and showed no cytotoxicity, which is similar to the results of this experiment (8). It has been reported in other contributions that iNOS is usually not expressed in animal tissues, but LPS stimulation causes upregulation of iNOS expression in mammary glands. In the current study, expression of iNOS may be closely related to the anti-inflammatory effect of ATC. We believe, based on the results of the current experiment, that ATC exerts anti-inflammatory function by downregulation of pro-inflammatory mediators. This downregulation may be attributed to the inhibition of the signal transduction pathways of TNF-α, IL-6, IL-1β, and iNOS expression. As a vital receptor for LPS recognition (43), TLR4 is able to activate the downstream MAPK signaling pathway (44). The MAPK signaling pathway involves ERK, JNK, and p38 kinases, and it plays an important role in the production of inflammatory factors, promoting the expression of pro-inflammatory cytokines (45). Our experimental results confirmed that ATC pretreatment inhibited LPS-induced TLR4 recognition and phosphorylation of downstream ERK and JNK, thereby inhibiting the expression of TNF-α, IL-6, IL-1β, and iNOS. In conclusion, our results clearly demonstrate that ATC exerts an anti-inflammatory effect by modulating the MAPK signal transduction pathway.

## Conclusion

Our results demonstrate that ATC pretreatment plays a role in anti-inflammatory action by interfering with TLR4 expression, which subsequently inhibits the downstream MAPK signaling pathways and the release of the pro-inflammatory cytokines TNF-α, IL-1β, and IL-6. Our findings provide the rationale and the mechanism for the prevention of bovine mastitis by ATC and suggest that ATC is an effective feed additive for the prevention and treatment of *E. coli*-induced mastitis.

## Authors’ contributions

Y.L. and X.J. contributed equally to this work, and they are both co-first authors. Y.L. and X.J. participated in the design of this study and performed most of the experiments. H.J.X. performed the Western blot and the real-time PCR experiments. J.Y.L., G.N.Z., X.J.D., and X.X.L. performed many assistants during all of the experiments. Y.L. performed the statistical analysis and drafted the main manuscript. Y.G.Z. supervised the work. Y.G.Z. revised the final version of the manuscript. All authors have read and approved the final version of the manuscript.
